# Time-dependent associations of documented low-dose aspirin administration with 28-day mortality and early safety outcomes in adults with sepsis: a MIMIC-IV cohort study

**DOI:** 10.3389/fmed.2026.1940916

**Published:** 2026-10-23

**Authors:** Chaoliang Hu, Dengwei Cheng, Shuli Hu

**Affiliations:** Department of Critical Care Medicine, Wuhan No. 1 Hospital, Wuhan, China

**Keywords:** aspirin, marginal structural model, MIMIC-IV, sepsis, time-dependent exposure

## Abstract

**Background:**

Associations between aspirin use and sepsis outcomes may reflect treatment timing and clinical selection.

**Methods:**

This retrospective Medical Information Mart for Intensive Care IV (MIMIC-IV) study included 31,888 adults with Sepsis-3. T0 was defined as the later of intensive care unit (ICU) admission or suspected infection; sepsis ascertainment could occur subsequently. Piecewise time-dependent Cox models evaluated actual enteral aspirin administration of 75–100 mg and 28-day restricted in-hospital and ICU mortality. Baseline sequential organ failure assessment (SOFA) measurements were restricted to T0 or earlier. Sensitivity analyses examined exposure history, eligibility timing, and a 24-h matched landmark cohort. A continuous-time marginal structural model (MSM) incorporated lagged clinical information. Modified Poisson models evaluated laboratory and transfusion outcomes.

**Results:**

Eligible aspirin administration was recorded at or after T0 for 4,771 patients, including 3,646 within 24 h. Adjusted in-hospital mortality hazard ratios (HRs) were 0.48 (95% CI 0.31–0.75) during 0–24 h and 0.63 (0.51–0.78) during 24–72 h, without clear associations thereafter. ICU estimates were similar. Qualifying SOFA scores occurred after T0 in 24,916 patients; restriction to the 6,972 patients with qualifying SOFA scores by T0 yielded HRs of 0.54 (0.28–1.03) and 0.62 (0.44–0.88) in the first two intervals. The MSM had residual imbalance and truncation-sensitive early estimates. After multiple imputation, the first two in-hospital HRs were 0.50 (0.32–0.79) and 0.66 (0.53–0.82), close to the primary estimates. A hemoglobin decline of ≥2 g/dL during 0–72 h was associated with early aspirin administration [adjusted risk ratio (RR) 1.10, 1.03–1.17], but 606 early recipients had a qualifying decline recorded before their first dose. The temporality-restricted 24–72-h analysis showed no clear association (RR 1.05, 0.91–1.21).

**Conclusions:**

Documented aspirin administration was associated with lower early restricted mortality. Retrospective eligibility, residual confounding, and uncertain prior exposure prevent causal interpretation; these findings do not justify initiating aspirin solely for sepsis.

## 1 Introduction

Sepsis is a life-threatening organ dysfunction caused by a dysregulated host response to infection and remains a major cause of critical illness and death worldwide (1, 2). Sepsis-3 remains the current international consensus case definition for adults. The 2025 Sequential Organ Failure Assessment (SOFA)-2 publication updated the organ-dysfunction score rather than defining Sepsis-4. The 2026 Surviving Sepsis Campaign revised the management recommendations but retained the Sepsis-3 framework (1, 3, 4). Sepsis-3 can be applied reproducibly in electronic health record studies, although future work should assess whether SOFA-2 changes cohort classification.

Aspirin inhibits platelet cyclooxygenase and thromboxane production. Its effects may extend to platelet activation and endothelial injury, with possible consequences for immunothrombosis and inflammation in sepsis (5–7). Several observational studies and meta-analyses have associated prehospital aspirin or antiplatelet therapy with lower sepsis mortality (8–13). Exposure definitions were not uniform. Some reports evaluated prior or continued therapy, whereas others included aspirin first given in the hospital.

Evidence from randomized trials has been less favorable. ANTISEPSIS found no reduction in sepsis-associated deaths with aspirin for primary prevention among healthy older adults (14). In a 2025 phase 2 trial, patients with established sepsis received 200 mg aspirin; organ dysfunction did not improve, and major bleeding was more frequent in the aspirin group (15). Safety findings should therefore be reported alongside mortality associations. Treatment selection patterns cannot establish efficacy.

Medication timing creates a separate source of bias. Classifying patients based on whether they ever receive aspirin assigns event-free time before the first dose to the exposed group and can create immortal time bias (16). Time-varying exposure aligns person-time with documented administration. It cannot, however, address bias when evolving platelet, coagulation, hemodynamic, and organ support variables influence subsequent aspirin administration and may themselves be affected by earlier treatment.

Using the Medical Information Mart for Intensive Care IV (MIMIC-IV) electronic Medication Administration Record (eMAR) data, this study evaluated the actual administration of single-ingredient enteral aspirin at 75–100 mg. The primary analysis estimated the association with 28-day-restricted in-hospital and intensive care unit (ICU) mortality over successive intervals after T0. Sensitivity analyses examined recorded aspirin history and the timing of sepsis ascertainment. A marginal structural model (MSM) incorporated measured, time-varying predictors of treatment. Hemoglobin decline, packed red blood cell (PRBC) administration, and invasive ventilation were assessed separately, with a focus on whether outcome events followed exposure.

## 2 Materials and methods

### 2.1 Study design, data source, and reporting

This retrospective cohort study used MIMIC-IV version 3.1 and linked MIMIC-IV-ED version 2.2 data (17, 18). Reporting followed the STrengthening the Reporting of OBservational studies in Epidemiology, the REporting of Studies Conducted using Observational Routinely Collected Health Data (RECORD), and the RECORD for pharmacoepidemiology (RECORD-PE) guidance (19–21). Analyses were conducted in R version 4.6.1 (R Foundation for Statistical Computing, Vienna, Austria). The source databases are de-identified. Their use was approved by the Institutional Review Boards of the Massachusetts Institute of Technology and Beth Israel Deaconess Medical Center, with a waiver of individual informed consent.

### 2.2 Cohort definition and index time

Adults with retrospectively ascertained Sepsis-3 were included, retaining the first eligible ICU stay per patient. A suspected infection had to occur no later than ICU discharge but could precede ICU admission. T0 was the later of ICU admission or suspected-infection onset. The derived Sepsis-3 definition linked suspected infection to a qualifying SOFA score of ≥2 within its permitted infection-related window. Because the SOFA score could occur after T0, this index was a retrospective temporal anchor, not proof that eligibility was established at the start of follow-up. Patients with missing or non-positive follow-up data were excluded. Sepsis-3 was used as the adult consensus definition; SOFA-2 updates organ dysfunction scoring rather than defining a replacement sepsis consensus (1, 4). Separate analyses restricted the cohort to T0 at ICU admission and to a qualifying SOFA score recorded by T0.

### 2.3 Aspirin exposure and pre-index aspirin evidence

The primary exposure was documented administration, not an order. eMAR records were screened for single-ingredient aspirin administration. Eligible administrations required a valid status and an oral or enteral route. Doses were restricted to 75–100 mg. Combination preparations, desensitization regimens, non-enteral administrations, orders without administration, and doses outside this range were excluded. In the time-dependent analyses, each patient remained unexposed until the exact first eligible administration and was considered exposed thereafter.

Recorded aspirin history was based on actual eligible administrations in the 24 h before T0. This variable and an active pre-index aspirin order indicator were both included in the baseline adjustment. The former also defined exposure-history strata. A further restriction excluded any recorded eligible aspirin administration from hospital admission to T0. Neither lookback establishes outpatient use or aspirin-naïve status. The original baseline eMAR variable represented any medication recorded in the pre-index 24-h window, not aspirin specifically. It was retained as a documentation-density covariate and relabeled accordingly.

### 2.4 Outcomes

The primary and secondary outcomes were in-hospital mortality and ICU mortality, both restricted to 28 days after T0. Follow-up ended at death, the relevant discharge, or 28 days. These are not all-cause 28-day mortality endpoints. Safety analyses examined hemoglobin decline of ≥2 g/dL and any PRBC administration through 72 h, comparing aspirin receipt within 24 h with no receipt in that window. Because the exposure and outcome windows overlap, temporally restricted analyses evaluated new events during 24–72 h among patients who were still being observed at 24 h without the corresponding earlier event. The hemoglobin analysis also required baseline and follow-up measurements. New invasive ventilation was assessed among patients without any baseline ventilatory support, with a corresponding 24–72-h analysis. These outcomes were not adjudicated for bleeding or acute respiratory distress syndrome (ARDS) diagnoses (Supplementary Methods).

### 2.5 Baseline covariates and missing data

Baseline clinical measurements used the 24-h pre-T0 window. The SOFA score was the latest assessment whose window ended no later than T0; a later Sepsis-3 qualifying score was not substituted. The covariates included demographics, time from ICU admission to T0, vital signs, laboratory measurements, SOFA score, comorbidities, ventilatory and vasopressor support, and the three medication record variables described above. Missing ICU vital signs were backfilled from linked emergency department records within the same window. Missing continuous measurements were median-imputed from observed baseline values with variable-specific missingness indicators. Table 1 reports observed values. Supplementary Table S1 and the Supplementary Methods give missingness and the full adjustment specification. For the sensitivity analysis, multiple imputation by chained equations (MICE) used predictive mean matching to impute 15 incomplete pre-index clinical measurements. The primary models were refitted in 20 imputed datasets without missingness indicators (Supplementary Methods S5).

**Table 1:** Baseline characteristics by eventual recorded eligible aspirin receipt.

Characteristics	No low-dose aspirin administration	Ever received low-dose aspirin	Absolute SMD
Patients, *n*	27,117	4,771	Not applicable
First eligible dose after T0, h	Not applicable	18.77 [10.94, 23.54]	Not applicable
Age, years	67.00 [55.00, 78.00]	71.00 [62.00, 79.00]	0.309
Male sex, *n* (%)	15,380 (56.7%)	3,043 (63.8%)	0.145
Time from ICU admission to T0, h	0.00 [0.00, 1.87]	0.00 [0.00, 4.33]	0.174
Heart rate, beats/min	89.41 [76.83, 104.33]	85.91 [75.00, 99.34]	0.175
Mean arterial pressure, mmHg	80.50 [71.50, 91.00]	80.00 [72.00, 90.50]	0.011
Respiratory rate, breaths/min	19.28 [16.50, 23.00]	20.00 [17.39, 23.20]	0.092
Temperature, °C	36.83 [36.48, 37.28]	36.84 [36.54, 37.28]	0.007
SpO_2_, %	97.59 [95.50, 99.36]	97.00 [95.20, 98.82]	0.102
Hemoglobin, g/dL	10.50 [9.00, 12.20]	10.60 [8.90, 12.50]	0.043
Platelet count, ×10^9^/L	188.00 [131.00, 260.00]	188.00 [141.00, 249.00]	0.031
White blood cell count, ×10^9^/L	11.30 [7.80, 16.00]	10.80 [7.70, 15.22]	0.065
Red cell distribution width, %	14.70 [13.60, 16.30]	14.50 [13.30, 16.20]	0.102
Creatinine, mg/dL	1.00 [0.70, 1.60]	1.10 [0.80, 1.90]	0.106
Blood urea nitrogen, mg/dL	21.00 [14.00, 35.00]	23.00 [16.00, 38.75]	0.064
Bicarbonate, mmol/L	23.00 [20.00, 26.00]	23.00 [20.00, 26.00]	0.011
International normalized ratio	1.30 [1.10, 1.60]	1.20 [1.10, 1.50]	0.087
Lactate, mmol/L	2.10 [1.40, 3.20]	1.80 [1.30, 2.60]	0.183
SOFA score	2.00 [1.00, 4.00]	4.00 [1.00, 6.00]	0.373
Congestive heart failure, *n* (%)	7,273 (26.8%)	2,347 (49.2%)	0.474
Chronic pulmonary disease, *n* (%)	7,005 (25.8%)	1,220 (25.6%)	0.006
Chronic renal disease, *n* (%)	5,679 (20.9%)	1,632 (34.2%)	0.300
Diabetes, *n* (%)	8,013 (29.5%)	1,941 (40.7%)	0.235
Malignant cancer, *n* (%)	4,131 (15.2%)	427 (8.9%)	0.194
Chronic liver disease, *n* (%)	4,507 (16.6%)	434 (9.1%)	0.226
Peptic ulcer disease, *n* (%)	910 (3.4%)	117 (2.5%)	0.054
Ventilator support, *n* (%)	6,463 (23.8%)	1,062 (22.3%)	0.037
Vasopressor support, *n* (%)	3,082 (11.4%)	658 (13.8%)	0.073
Pre-index active aspirin order, *n* (%)	4,504 (16.6%)	1,519 (31.8%)	0.361
Recorded eligible aspirin administration in the 24 h before T0, *n* (%)	339 (1.3%)	1,460 (30.6%)	0.876
Any medication eMAR record in the 24-h pre-index window, *n* (%)	9,891 (36.5%)	4,218 (88.4%)	1.270

Values are observed as median [IQR] or *n* (%). Eventual receipt is descriptive, not a baseline treatment assignment. The SOFA score only uses assessments ending by T0. The availability of any medication eMAR is not aspirin-specific. Missingness is presented in Supplementary Table S1.

### 2.6 Primary time-dependent analysis

The Cox counting process models used robust standard errors clustered by ICU stay (22). Aspirin status changed at the first eligible dose. The primary model estimated separate hazard ratios (HRs) during 0 to <24, 24 to <72, 72 to <168, and 168 h to 28 days, with baseline hazards stratified by interval. These intervals were measured from T0, not from the first dose. Natural cubic splines with 3 degrees of freedom were used for age, admission year, heart rate, mean arterial pressure (MAP), platelet count, creatinine, and lactate. Other terms were linear or categorical as appropriate. The constant-effect formulation was not used for the principal clinical interpretation.

### 2.7 Marginal structural model

The inverse-probability-weighted MSM sensitivity analysis used continuous-time initiation weights (23, 24). The first eligible administration was modeled for up to 28 days of untreated ICU observation, including initiations after 72 h. Piecewise exponential rate models retained the actual risk duration and the exact first-dose times. Day-0 covariates were strictly pre-index, including observed baseline PRBC use. Later days used prior-day clinical measurements and organ-support indicators. Missing updates were carried forward, followed by baseline-only median fallback when needed. Three first doses recorded exactly at T0 were excluded from the MSM only. The detailed model specification appears in the Supplementary Methods.

The numerator rate model included time blocks; the denominator also included baseline variables and lagged predictors of administration. Predictable weights accumulated continuously before initiation, changed by the numerator-to-denominator rate ratio at initiation, and remained fixed thereafter or after ICU observation ended. Weighted piecewise Cox models used hourly weight updates and exact exposure times; half-hour updates checked numerical sensitivity. Primary weights were truncated at the 1st/99th percentiles, with raw and 2.5th/97.5th-percentile alternatives. Diagnostics included weight distributions, risk-record effective sample sizes, model convergence, and standardized initiation process balance. No probability clipping was used. Discharge was the operational endpoint, not a censoring event adjusted by an additional weight model.

### 2.8 Landmark analysis

The 24-h landmark analysis required survival and continued hospital and ICU observation beyond 24 h (25). Exposure was eligible aspirin during the initial 24 h. Propensity scores used the baseline covariates, including recorded pre-index aspirin and missingness indicators. Nearest neighbor matching was 1:1, with a caliper of 0.1 SD on the estimated propensity score scale, exact matching on congestive heart failure, and Mahalanobis matching on age (26, 27). Controls outside common support were discarded, and propensity scores were re-estimated. Continuous terms were linear in the propensity and adjusted landmark models. An absolute standardized mean difference (SMD) <0.10 was the balance threshold (28). The Cox models were clustered by matched subclass and estimated post-landmark associations, conditional on survival and continued observation.

### 2.9 Additional sensitivity, subgroup, and safety analyses

Piecewise models were stratified by recorded eligible aspirin administration in the 24 h before T0, with a joint test across interval-specific interactions. Additional restrictions required no recorded aspirin administration since admission, T0 at ICU admission, or a qualifying SOFA score by T0. Observed baseline platelet count <100 vs. ≥100 × 10^9^/L defined an exploratory subgroup, not sepsis-induced thrombocytopenia or disseminated intravascular coagulation (DIC). Modified Poisson regression with robust HC0 variance estimated the adjusted risk ratios (RRs) for the safety and respiratory outcomes (29). The restricted, subgroup, interaction, and Poisson models used the same baseline variables as the primary model, but MAP was linear rather than a spline; constant terms were omitted. A two-sided *P* < 0.05 was the nominal significance threshold. No adjustment for multiple comparisons was applied to these exploratory analyses.

## 3 Results

### 3.1 Cohort and exposure

Among 31,910 extracted records, 22 were excluded before analysis (4 with hospital death but missing death time and 18 with non-positive hospital follow-up), leaving 31,888 patients (Figure 1). Eligible low-dose aspirin use was recorded at or after T0 through the documented ICU stay of 4,771 patients, including 3,646 within 24 h. Six first records occurred at or after 28 days and one after recorded death; these did not contribute exposed person-time to the corresponding mortality models. The median time to the first recorded eligible administration was 18.77 h [interquartile range (IQR) 10.94–23.54] (Figure 2). Patients with eventual recorded receipt were older and had more cardiovascular and renal comorbidities (Table 1). Actual eligible aspirin administration in the 24 h before T0 was recorded in 339/27,117 patients (1.3%) without subsequent recorded receipt and 1,460/4,771 patients (30.6%) with subsequent receipt.

**Figure 1:**
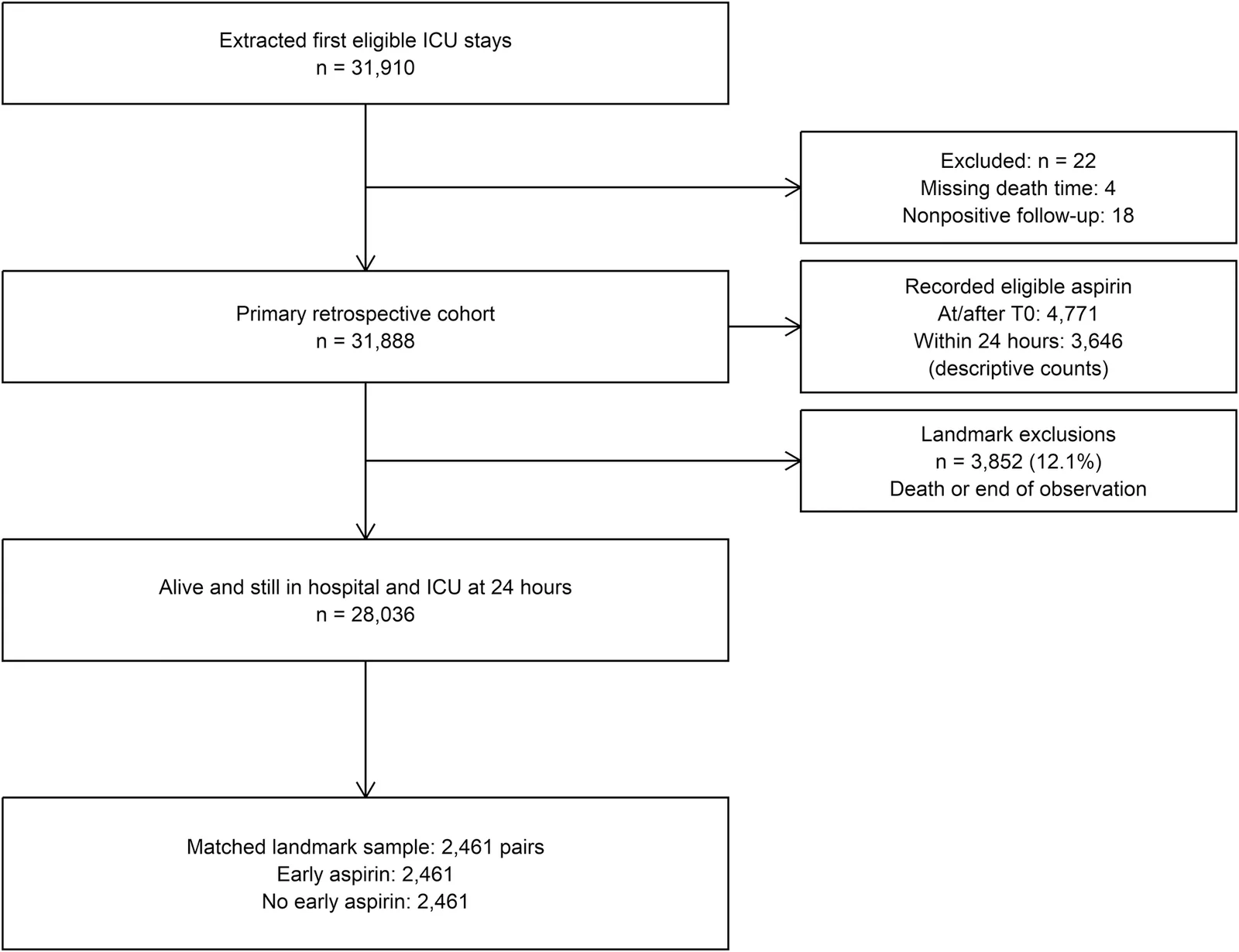
Cohort assembly and 24-h landmark analysis. Of the 31,888 patients in the primary cohort, 28,036 survived and remained under both hospital and ICU observation beyond 24 h; of these, 3,852 (12.1%) were excluded from the landmark analysis. The early death and discharge categories overlap and are detailed in Supplementary Table S4. The side box containing the exposure summary describes eligible low-dose aspirin administration at or after T0 through the recorded ICU stay and is not a landmark stage.

**Figure 2:**
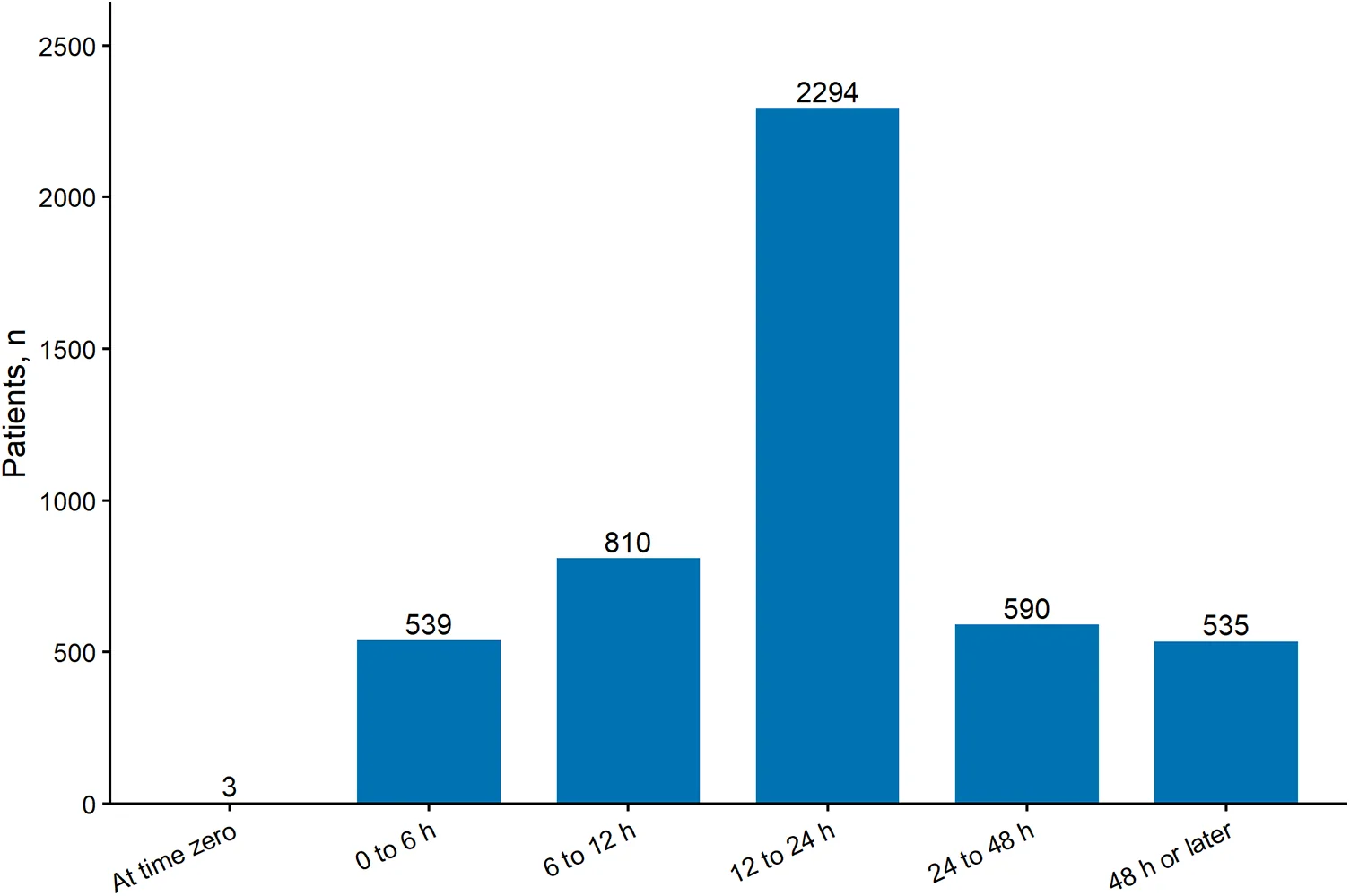
Timing of the first eligible low-dose aspirin administration. Eligible exposure required the actual administration of single-ingredient enteral aspirin 75–100 mg.

### 3.2 Primary time-dependent mortality analysis

There were 5,208 in-hospital and 3,792 ICU deaths within the restricted 28-day follow-up. The adjusted in-hospital mortality HRs were 0.48 (0.31–0.75), 0.63 (0.51–0.78), 0.87 (0.74–1.02), and 0.94 (0.82–1.07) across the four intervals, respectively. The first two estimates were below 1.00 (*P* = 0.001 and *P* < 0.001); later intervals showed no clear association. The corresponding ICU HRs were 0.49 (0.31–0.77), 0.65 (0.52–0.80), 0.91 (0.76–1.10), and 1.07 (0.90–1.28), respectively (Table 2; Figure 3). These estimates describe associations with documented administration, not randomized treatment effects. In the MICE analysis, the first two in-hospital HRs were 0.50 (0.32–0.79) and 0.66 (0.53–0.82); the ICU HRs were 0.51 (0.32–0.80) and 0.67 (0.54–0.84) (Supplementary Table S9).

**Table 2:** Adjusted piecewise time-dependent mortality associations.

Outcome	Interval after T0	No. of patients	No. of deaths	HR (95% CI)	*P*-value
In-hospital	0 to <24 h	31,888	5,208	0.48 (0.31–0.75)	0.001
In-hospital	24 to <72 h	31,888	5,208	0.63 (0.51–0.78)	<0.001
In-hospital	72 to <168 h	31,888	5,208	0.87 (0.74–1.02)	0.083
In-hospital	168 h to 28 days	31,888	5,208	0.94 (0.82–1.07)	0.336
ICU	0 to <24 h	31,888	3,792	0.49 (0.31–0.77)	0.002
ICU	24 to <72 h	31,888	3,792	0.65 (0.52–0.80)	<0.001
ICU	72 to <168 h	31,888	3,792	0.91 (0.76–1.10)	0.319
ICU	168 h to 28 days	31,888	3,792	1.07 (0.90–1.28)	0.431

The patient and death counts are full-cohort totals, not interval-specific risk sets. The models use exact first dose times and baseline adjustment. Discharge ends the relevant mortality follow-up.

**Figure 3:**
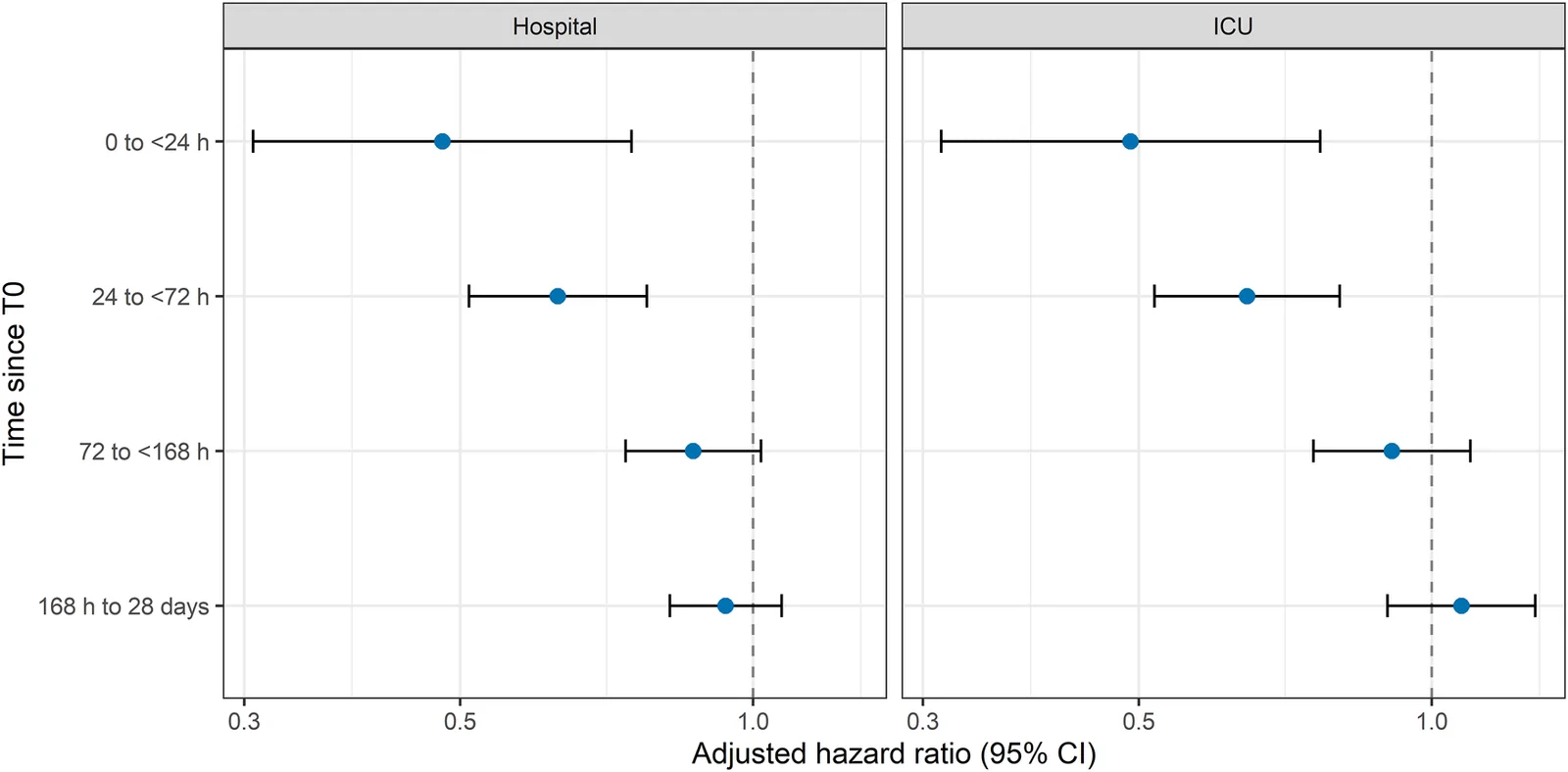
Time-dependent aspirin associations with restricted in-hospital and ICU mortality. Intervals are measured from T0, not from the first dose. Points indicate adjusted HRs, horizontal bars 95% CIs, and the dashed vertical line HR = 1.00.

### 3.3 Exposure history sensitivity analysis

Among early aspirin recipients, 1,330/3,646 (36.5%) had an actual eligible administration recorded in the 24 h before T0. For patients without such a record (*n* = 30,089), the in-hospital HRs were 0.46 (0.26–0.79), 0.64 (0.50–0.83), 0.79 (0.65–0.96), and 1.01 (0.88–1.16), respectively; for those with a record (*n* = 1,799), they were 0.40 (0.17–0.97), 0.43 (0.24–0.76), 0.85 (0.48–1.49), and 1.04 (0.52–2.05), respectively. The global interaction *P*-values were 0.122 for in-hospital mortality and 0.393 for ICU mortality. Excluding the 2,211 patients with recorded eligible aspirin administration from hospital admission to T0 left 29,677 patients, with in-hospital HRs of 0.38 (0.20–0.70), 0.60 (0.46–0.79), 0.79 (0.64–0.96), and 0.98 (0.85–1.13), respectively (Supplementary Table S2). A broader lookback still cannot identify verified *de novo* use.

### 3.4 Marginal structural model

The MSM included 31,885 patients and 150,256 daily records of untreated time at risk for aspirin initiation (including records shorter than 24 h), with 4,761 observed initiations, including 378 at or after 72 h. Raw weights had a mean of 0.98, an SD of 0.49, a median of 0.90, a 99th percentile of 2.26, and a maximum of 32.50. Weight truncation affected 3,006 of these daily records and increased the record-level effective sample size from 120,120 to 142,609. The implied denominator, the 24-h initiation probability, was <0.01 for 90,288 records. After truncation, the largest residual standardized initiation-process difference was 0.335. Treatment overlap remained limited, and balance was incomplete. Supplementary Figure S2 compares the raw and truncated weight distributions.

After 1st/99th-percentile truncation, the in-hospital HRs were 0.38 (0.22–0.68), 0.55 (0.42–0.72), 0.90 (0.74–1.09), and 1.02 (0.89–1.18), respectively; the ICU HRs were 0.39 (0.22–0.69), 0.59 (0.45–0.78), 0.95 (0.76–1.19), and 1.10 (0.91–1.33), respectively (Table 3; Supplementary Table S8). Without truncation, the first-interval estimates were 0.70 (0.17–2.83) for in-hospital mortality and 0.72 (0.18–2.89) for ICU mortality. Because the early estimates changed with truncation, the MSM does not independently confirm an early treatment effect.

**Table 3:** Adjusted safety, respiratory, and MSM sensitivity analyses.

Outcome	Contrast or interval	No. of patients	No. of events	Effect (95% CI)	*P*-value
Hemoglobin decline, 0–72 h	Early aspirin vs. no early aspirin	16,410	5,313	RR 1.10 (1.03–1.17)	0.004
Incident hemoglobin decline, 24–72 h	Early aspirin vs. no early aspirin	9,947	2,013	RR 1.05 (0.91–1.21)	0.502
Any PRBC, 0–72 h	Early aspirin vs. no early aspirin	31,888	7,759	RR 0.88 (0.82–0.96)	0.003
Any PRBC, 24–72 h	Early aspirin vs. no early aspirin	22,693	1,948	RR 0.84 (0.70–1.00)	0.046
New invasive ventilation, 0–72 h	Early aspirin vs. no early aspirin	24,363	10,314	RR 0.99 (0.94–1.04)	0.723
New invasive ventilation, 24–72 h	Early aspirin vs. no early aspirin	12,276	954	RR 0.82 (0.65–1.05)	0.116
In-hospital mortality	MSM; 0 to <24 h	31,885	5,208	HR 0.38 (0.22–0.68)	<0.001
In-hospital mortality	MSM; 24 to <72 h	31,885	5,208	HR 0.55 (0.42–0.72)	<0.001
In-hospital mortality	MSM; 72 to <168 h	31,885	5,208	HR 0.90 (0.74–1.09)	0.276
In-hospital mortality	MSM; 168 h to 28 days	31,885	5,208	HR 1.02 (0.89–1.18)	0.747
ICU mortality	MSM; 0 to <24 h	31,885	3,792	HR 0.39 (0.22–0.69)	0.001
ICU mortality	MSM; 24 to <72 h	31,885	3,792	HR 0.59 (0.45–0.78)	<0.001
ICU mortality	MSM; 72 to <168 h	31,885	3,792	HR 0.95 (0.76–1.19)	0.679
ICU mortality	MSM; 168 h to 28 days	31,885	3,792	HR 1.10 (0.91–1.33)	0.318

Early exposure is defined as receipt within 24 h. Concurrent 0–72-h outcomes may precede treatment. The 24–72-h analyses require survival, continued observation, and absence of the relevant earlier event; the hemoglobin analysis also requires repeated measurements. RRs use modified Poisson regression with HC0 variance. MSM HRs use 1st/99th-percentile-truncated weights. A CI endpoint rounded to 1.00 may coexist with *P* < 0.05. PRBC estimates do not establish protection from bleeding.

### 3.5 Landmark analysis and early exclusions

At 24 h, 28,036 patients (87.9%) were alive and still under observation in the hospital and ICU. The remaining 3,852 (12.1%) were excluded. During those 24 h, 698 patients died in the hospital, and 689 died in the ICU; 3,149 left the ICU alive, and 142 were discharged from the hospital alive. These categories overlap and should not be added together. A total of 2,461 early aspirin recipients were matched to 2,461 comparators; the maximum absolute SMD was 0.079 (Figure 4; Supplementary Figure S1). The adjusted post-landmark HR was 0.81 (0.70–0.94) for in-hospital mortality and 0.86 (0.72–1.03) for ICU mortality (Table 4). These estimates pertain only to matched 24-h survivors who remained under observation.

**Figure 4:**
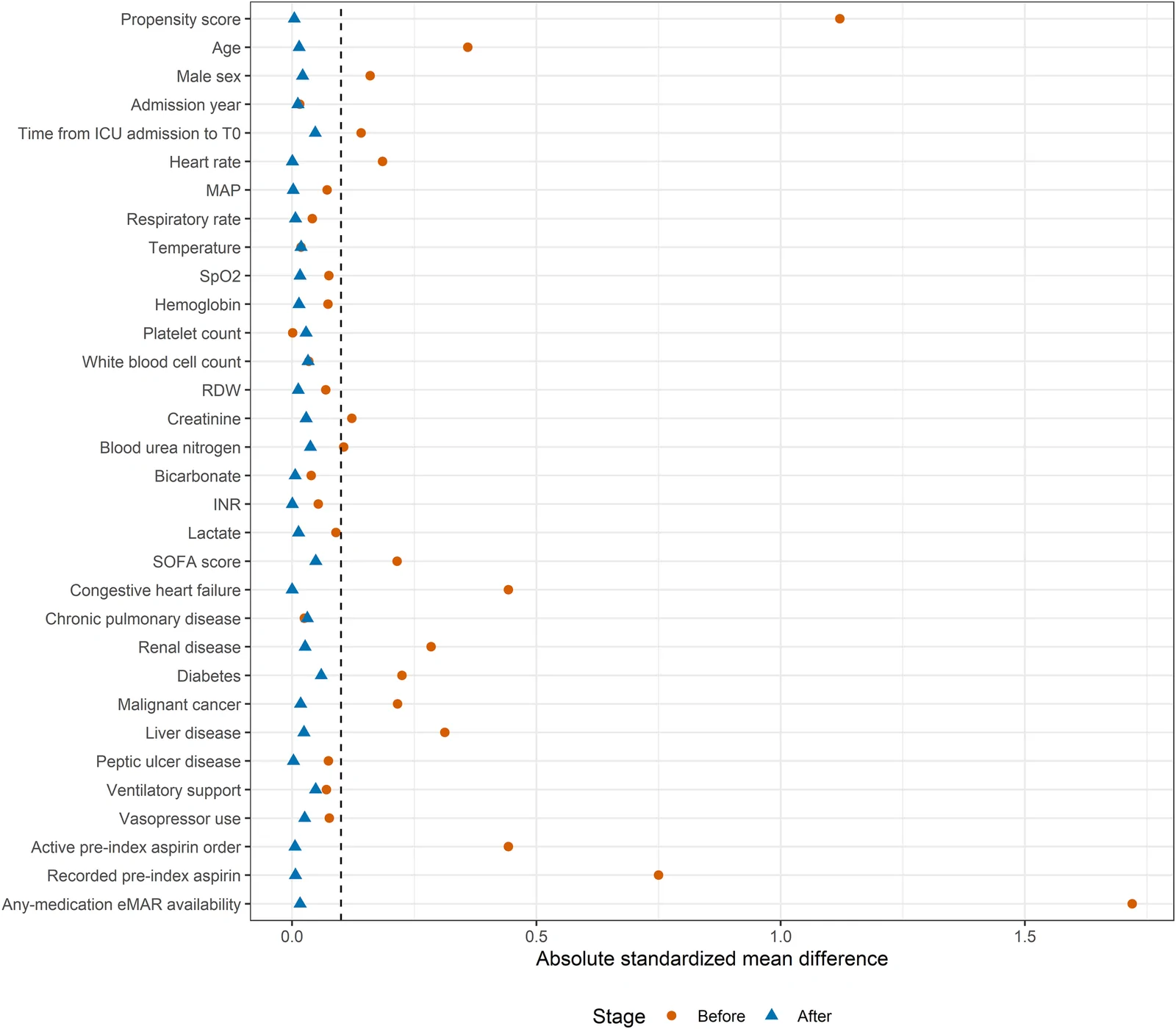
Covariate balance before and after the 24-h landmark matching of 2,461 pairs. Symbols indicate absolute SMDs; the dashed line marks 0.10. Continuous matching covariates were median-imputed, with missingness-indicator balance shown in Supplementary Figure S1.

**Table 4:** Twenty-four-hour propensity-matched landmark analysis.

Outcome	Model	No. of patients	No. of deaths	HR (95% CI)	*P*-value
In-hospital	Matched	4,922	760	0.83 (0.72–0.95)	0.009
In-hospital	Matched + adjusted	4,922	760	0.81 (0.70–0.94)	0.005
ICU	Matched	4,922	546	0.90 (0.76–1.07)	0.239
ICU	Matched + adjusted	4,922	546	0.86 (0.72–1.03)	0.099

The sample comprises 2,461 matched pairs who survived and remained in the hospital and ICU beyond 24 h. Standard errors are clustered by matched subclass. These estimates are conditional on landmark selection.

### 3.6 T0 timing and platelet subgroup analyses

T0 coincided with ICU admission in 16,845 patients (52.8%). The delay from ICU admission to T0 was >0–6 h in 10,754 patients (33.7%), >6–24 h in 1,613 patients (5.1%), >24–48 h in 783 patients (2.5%), and >48 h in 1,893 patients (5.9%). When T0 was restricted to ICU admission, the in-hospital HRs in the first two intervals were 0.43 (0.22–0.84) and 0.54 (0.40–0.74), respectively. The qualifying Sepsis-3 SOFA score was recorded after T0 in 24,916 patients (78.1%). Among the 6,972 patients whose qualifying SOFA was available by T0, the in-hospital HRs were 0.54 (0.28–1.03), 0.62 (0.44–0.88), 0.82 (0.63–1.06), and 0.76 (0.60–0.96), respectively. The first interval was imprecise, and the later pattern differed from that of the full cohort (Supplementary Table S5).

An observed baseline platelet count was available for 16,892 patients; 2,260 had <100 × 10^9^/L. The global interaction *P*-values were <0.001 for in-hospital mortality and 0.020 for ICU mortality (Supplementary Table S6). Approximately 47% of the cohort lacked an observed pre-index platelet count. This selected, exploratory analysis does not establish the benefits or risks of treating patients with sepsis-induced thrombocytopenia or DIC.

### 3.7 Hemoglobin, PRBCs, and respiratory outcomes

Among the 16,410 patients with evaluable 0–72-h hemoglobin measurements, a decline of ≥2 g/dL occurred in 963/2,377 early aspirin recipients (40.5%) and 4,350/14,033 comparators (31.0%). The adjusted RR was 1.10 (95% CI 1.03–1.17; *P* = 0.004). A qualifying decline was already documented before the first dose in 606 early recipients. In the temporality-restricted 24–72-h sample (*n* = 9,947), events occurred in 257/1,317 early recipients and 1,756/8,630 comparators; the adjusted RR was 1.05 (0.91–1.21; *P* = 0.502). PRBC administration through 72 h occurred in 717/3,646 early recipients (19.7%) and 7,042/28,242 comparators (24.9%), with an adjusted RR of 0.88 (0.82–0.96; *P* = 0.003). Among the 22,693 patients observed beyond 24 h without earlier PRBC administration, the 24–72-h RR was 0.84 (0.70–1.00; *P* = 0.046). Lower PRBC estimates may reflect withholding aspirin in patients at higher bleeding risk and were not interpreted as protective.

Among the 24,363 patients without baseline ventilatory support, 10,314 received invasive ventilation through 72 h. The adjusted RR was 0.99 (0.94–1.04; *P* = 0.723). Among the 12,276 patients still observed after 24 h without baseline or first-24-h ventilation, the 24–72-h RR was 0.82 (0.65–1.05; *P* = 0.116) (Supplementary Table S7). Neither result provided clear evidence of an association. This operational endpoint was not treated as a validated ARDS phenotype.

## 4 Discussion

Documented low-dose enteral aspirin administration was associated with lower restricted in-hospital and ICU mortality during the first 72 h after T0 in the primary models. Later estimates were less precise and closer to 1.00. Restriction to patients whose qualifying SOFA score was already recorded by T0 did not reproduce every interval-specific finding. The MSM also showed residual imbalance and sensitivity to weight truncation. The early HRs changed little after MICE. This analysis assumes missingness at random, a condition that cannot be verified. These results leave substantial uncertainty about treatment selection. The concurrent 0–72-h hemoglobin association was attenuated in an analysis that required the outcome to occur after the exposure window.

The mortality estimates point in the same direction as earlier observational studies on prehospital or chronic aspirin administration and recent meta-analyses (8–13). In a 2024 cohort, the continuation of chronic antiplatelet therapy was associated with survival, while the difference in transfusion was not statistically clear (30). A 2026 MIMIC-IV/eICU study of patients with atrial fibrillation also linked early aspirin administration to lower mortality. The association was more apparent among presumed new starters, although external cohort estimates were attenuated (31). Similar findings across these studies may reflect a biological association, but common sources of bias, such as treatment selection, medication continuation, contraindications, and underlying health status, remain plausible.

Randomized findings argue against an efficacy interpretation. In the 2025 phase 2 trial, 200 mg aspirin did not improve the SOFA trajectory, and major bleeding was more frequent with aspirin (15). A 2026 meta-analysis judged the evidence on mortality and bleeding to be of very low certainty and did not recommend the routine use of aspirin for sepsis (13). Its dose, population, exposure definition, and estimand differ from those of the present study, which limits direct comparison. An inverse association in database analyses does not constitute treatment advice.

Inpatient aspirin records could not establish whether the therapy had been used chronically before admission. The original eMAR variable captured documentation of any medication rather than aspirin itself. Eligible aspirin administration before T0 was recorded in 36.5% of early recipients. Mortality was examined within the resulting history strata and after exclusion of every eligible aspirin record since hospital admission. Neither analysis verifies home medication use. Healthy user and continuation biases remain possible, and the absence of an inpatient aspirin record does not mean that a patient was aspirin-naïve.

The timing of the hemoglobin decline changes how the crude contrast should be read. Many qualifying declines occurred before the first aspirin dose; therefore, subsequent administration cannot explain the 0–72-h association. No clear association was seen during the 24–72 h, but the confidence interval and sample restrictions do not establish safety. PRBC use did not increase. This comparison may be affected by transfusion practices, non-bleeding causes of hemoglobin decline, and the decision to withhold aspirin. Prospective studies should adjudicate major bleeding and record the indication for transfusion. Randomized aspirin data also support explicit bleeding surveillance (15, 32).

Using actual eMAR times avoided classifying unfilled orders as treatment and retained unexposed person-time before the first dose. The temporal audit kept the baseline SOFA separate from the qualifying assessments recorded later. Sensitivity analyses examined recorded treatment history and eligibility timing; landmark selection and alternative weight truncation were also assessed. The continuous-time initiation model used dose timing together with prior clinical measurements. The code and aggregate outputs are available.

The main cohort was assembled retrospectively. Qualifying SOFA scores were recorded after T0 in 78.1% of patients, which means that sepsis eligibility had not been established when follow-up began. Selection based on subsequent organ dysfunction may persist even when exposure is timed exactly and an MSM is used. Restriction to patients with a qualifying SOFA score by T0 markedly reduced the cohort and left imprecise early estimates. Confounding by treatment indication also remains possible. Limited treatment overlap and the sensitivity of MSM estimates to truncation further restrict causal interpretation. The landmark analysis excluded 12.1% of the original cohort and applies only to selected survivors. For patients whose T0 followed ICU admission, baseline physiology may have already reflected ICU treatment. SOFA-2 assigns different points for respiratory, cardiovascular, and kidney dysfunction to account for contemporary organ support (4). Rescoring could alter the baseline severity strata and the severity adjustment in the mortality models. If SOFA-2 were used for exploratory case ascertainment, the patients meeting organ-dysfunction criteria and the time at which they qualify might also change. Timing is relevant here because qualifying assessments based on the original SOFA often occurred after T0. The Sepsis-3 ≥2-point increase was defined using the original SOFA (1); applying that threshold to SOFA-2 would require validation. Because SOFA-2 was not reconstructed for this cohort, the direction and magnitude of any change in eligibility or adjusted associations remain unknown.

A strictly pre-index SOFA value was unavailable for 20,784 patients (65.2%). Baseline platelet count was missing for 47.0% of patients, and lactate was missing for 71.3%. Median imputation with missingness indicators may leave residual confounding and does not account for uncertainty in the imputed values. MICE gave similar interval-specific estimates, but it relies on a missing-at-random assumption that cannot be verified. Neither method can eliminate bias from unmeasured severity or informative missingness (33). The platelet analysis cannot define a DIC phenotype. Because observations ended at discharge, the mortality models could not estimate all-cause 28-day mortality or a competing-risk cumulative incidence. The safety and respiratory outcomes were unadjudicated surrogates and were incompletely measured. As this is a single-center study, the results may not apply to other settings, doses, or patients with specific contraindications.

The findings leave the clinical equipoise unchanged and do not support the routine aspirin use for sepsis treatment. A future target trial would need to separate verified chronic continuation from randomized initiation. Patients at high risk of major bleeding should be stratified or excluded. Updated organ dysfunction measures, including SOFA-2, should also be evaluated. Mortality, bleeding, transfusion, and respiratory outcomes require prospective adjudication (4, 34, 35).

## 5 Conclusions

Documented low-dose aspirin administration was associated with lower early restricted mortality. Retrospective eligibility and residual treatment-selection bias preclude causal inference, and the analysis does not establish bleeding safety or support aspirin initiation solely for sepsis.

## Data Availability

The raw data supporting the conclusions of this article will be made available by the authors, without undue reservation.
