# Supplementary material for: Time-dependent associations of documented low-dose aspirin administration with 28-day mortality and early safety outcomes in adults with sepsis: a MIMIC-IV cohort study
Source: Front Med (Lausanne). 2026 Oct 23;13:1940916. doi: 10.3389/fmed.2026.1940916 (PMC13739798; doi:10.3389/fmed.2026.1940916)
Supplement: Supplementary file 1 [file Supplementaryfile1.docx]

Supplementary Material

# Supplementary methods

## S1 Exposure history and baseline adjustment

Eligible aspirin was single-ingredient enteral aspirin 75–100 mg with documented actual administration. The 24-hour pre-index indicator, the admission-to-T0 administration lookback, and the active-order variable measure different forms of inpatient evidence. None verifies chronic home use. The any-medication eMAR variable measures documentation density.

T0 remains the later of ICU admission or suspected infection. Sepsis-3 ascertainment is retrospective, and the qualifying SOFA assessment may follow T0. Baseline SOFA instead uses the latest assessment ending in the 24 hours up to T0; no later qualifying score is substituted. Missing SOFA was imputed to the observed baseline median of 2 with a missingness indicator. Restriction to qualifying SOFA by T0 addresses one aspect of eligibility timing but is not a new primary cohort.

The adjustment set included age, sex, admission year, hours from ICU admission to T0, heart rate, MAP, respiratory rate, temperature, SpO₂, hemoglobin, platelet count, white blood cell count, red cell distribution width, creatinine, blood urea nitrogen, bicarbonate, INR, lactate, and SOFA. Comorbidities comprised congestive heart failure, chronic pulmonary disease, renal disease, diabetes, malignant cancer, liver disease, and peptic ulcer disease. Baseline ventilatory support, vasopressors, active aspirin order, actual eligible aspirin in the previous 24 hours, and any-medication eMAR availability were also included. Each of the 15 clinical measurements had a missingness indicator; indicators that were constant in a restricted sample were omitted. PT and PTT were not adjustment variables.

Primary Cox models used natural cubic splines with 3 degrees of freedom for age, admission year, heart rate, MAP, platelet count, creatinine, and lactate. Restricted, subgroup, interaction, and modified Poisson models used the same variables, but MAP was linear; other spline terms were unchanged. Stratifying variables and other constant terms were omitted where appropriate. Landmark propensity and covariate-adjusted outcome models used linear continuous terms and the same available baseline variables. The interaction tests were joint robust Wald tests of four exposure-by-subgroup terms.

## S2 Safety and respiratory outcomes

Early exposure meant an eligible dose during the first 24 hours. The 0–72-hour hemoglobin outcome was a decline ≥2 g/dL from the observed pre-T0 baseline to the lowest ICU measurement through 72 hours. This concurrent window can include declines before the first dose. A separate 24–72-hour analysis required survival and continued hospital/ICU observation at 24 hours, an observed baseline value, measurements in both 0–24 and 24–72 hours, and no decline ≥2 g/dL during 0–24 hours. Its outcome was the first qualifying decline during 24–72 hours relative to the original baseline. Selection by survival and measurement availability remains possible.

PRBC events were taken from mimiciv_icu.inputevents using locally checked item IDs 220996, 225168, 226368, and 227070. Rewritten records and nonpositive amounts were excluded. Event start times defined any PRBC administration in T0–72 hours and baseline PRBC in the 24 hours before T0. The 24–72-hour analysis required continued ICU/hospital observation beyond 24 hours and no PRBC event during 0–24 hours. These outcomes used documented administration, not adjudicated bleeding; heterogeneous volumes were not converted into units.

New invasive ventilation was evaluated among patients without baseline ventilatory support of any type. The 24–72-hour analysis additionally required continued observation at 24 hours and no invasive ventilation in the first 24 hours. Invasive ventilation was an operational respiratory-support outcome, not an ARDS diagnosis. Modified Poisson models used HC0 sandwich variance and the adjustment specification above.

## S3 Continuous time marginal structural model

The MSM modeled the first eligible post-T0 administration while patients remained untreated and under ICU observation, for up to 28 days. Three patients whose first dose was exactly at T0 were excluded from this sensitivity analysis because an instantaneous baseline treatment mass is not represented by a continuous initiation intensity. They remained in the primary analysis. Piecewise-exponential Poisson models used actual untreated risk duration as an offset and retained exact initiation times. Time blocks were individual days 0–6, followed by days 7–13, 14–20, and 21–27. The numerator contained time blocks only. The denominator contained the baseline adjustment variables, baseline PRBC use, and prior-day hemoglobin, platelet count, INR, lactate, creatinine, MAP, SOFA, ventilatory support, vasopressors, and PRBC use. Interactions linked log(day+1) to lagged platelet count, INR, MAP, SOFA, and recorded pre-index aspirin.

Baseline values were available no later than T0. Later days used the latest laboratory and SOFA measurements from the preceding day, mean prior-day MAP, and any prior-day organ-support or PRBC event. Missing updates were carried forward, with baseline-only medians used when no earlier measurement existed. Missing-update indicators were retained. Continuous predictors were winsorized at baseline 1st/99th-percentile thresholds and scaled using baseline values; those thresholds also applied to the corresponding lagged measurements. Day-0 PRBC status came from actual pre-index events, rather than an assumed absence of transfusion.

For each patient, the weight before initiation was exp of the cumulative integral of the denominator initiation rate minus the numerator rate. At the exact first dose it was multiplied by the numerator-to-denominator rate ratio. The weight was then held constant; it was also held constant after ICU observation ended if no dose had occurred. This continuous-time likelihood ratio avoids assigning a full day’s observed treatment status to earlier person-time. Weighted Cox models evaluated weights at the start of hourly subintervals and split exposure at the exact first dose. A half-hour grid checked numerical sensitivity. Primary truncation used the 1st/99th percentiles of weights at the ends of untreated risk-day records; Supplementary Figure S2 shows the distribution of these weights before and after truncation. Untruncated and 2.5th/97.5th-percentile results were also reported. No predicted-probability clipping was applied. Standard errors were clustered by ICU stay. No additional censoring model was fitted; discharge ended the operational in-hospital or ICU mortality endpoint.

Diagnostics report risk-record weight distributions and effective sample sizes, not patient-level effective sample sizes. Initiation-process balance compares covariate means among weighted observed initiations with means in marginal-intensity-weighted untreated risk time, standardized by the pooled weighted standard deviation. It is not an ordinary treated-versus-untreated daily SMD. Raw risk-time integrals were evaluated analytically, and truncated integrals used 24 midpoint subdivisions per risk-day record. Residual differences and truncation sensitivity were retained as limitations rather than treated as proof of successful confounding control.

## S4 Other sensitivity analyses

The landmark sample required hospital and ICU follow-up beyond 24 hours. Propensity scores were re-estimated after discarding controls outside common support, then matched 1:1 with a 0.1-SD caliper on the propensity-score scale, exact congestive-heart-failure matching, and Mahalanobis age matching. The outcome models used matched-subclass robust variance. The platelet subgroup used observed counts <100 versus ≥100×10^9/L and did not diagnose DIC. Restricted and subgroup analyses were exploratory and not adjusted for multiplicity.

Numerical validation passed likelihood-path and integration checks. Halving the outcome-weight grid from 1 hour to 0.5 hour changed any primary truncated MSM HR by at most 0.0001 in absolute terms (0.02% relative change). This numerical check does not validate the causal assumptions.

## S5 Multiple-imputation sensitivity analysis

Twenty imputed data sets were generated with the mice package (version 3.19.0) using five chained-equation iterations and predictive mean matching with five donors. Imputation was confined to the 15 incomplete clinical measurements observed no later than T0, including platelet count, lactate, and strict pre-index SOFA. Other baseline covariates were considered for the imputation model. Auxiliary predictors included hospital and ICU mortality, log-restricted follow-up times, recorded aspirin receipt, early receipt, and log first-dose time. Predictor selection required a minimum absolute correlation of 0.08 and a minimum proportion of usable cases of 0.20; age, sex, admission year, and the outcome and treatment auxiliaries were always included. Three implausible observed MAP values above 300 mmHg were set to missing for this sensitivity analysis; the primary model was unchanged. The completed-data Cox models retained the original exact-time exposure definition, interval-specific hazards, splines, and cluster-robust variance. Missingness indicators were omitted. Log HRs and robust variances were pooled by Rubin's rules. This analysis assumes missingness at random conditional on the included variables and cannot address unmeasured severity, missing-not-at-random mechanisms, or retrospective eligibility.

**Supplementary Table S1. Missing pre-index clinical measurements.**

| **Variable** | **Missing, n** | **Missing, %** |
| --- | --- | --- |
| Heart rate | 10,945 | 34.3% |
| Mean arterial pressure (MAP) | 11,681 | 36.6% |
| Respiratory rate | 10,199 | 32.0% |
| Temperature | 13,177 | 41.3% |
| SpO₂ | 11,372 | 35.7% |
| Hemoglobin | 15,149 | 47.5% |
| Platelet count | 14,996 | 47.0% |
| White blood cell count | 15,213 | 47.7% |
| Red cell distribution width | 15,245 | 47.8% |
| Creatinine | 15,680 | 49.2% |
| Blood urea nitrogen | 15,705 | 49.3% |
| Bicarbonate | 15,909 | 49.9% |
| INR | 18,097 | 56.8% |
| Lactate | 22,746 | 71.3% |
| SOFA score | 20,784 | 65.2% |

Denominator: 31,888. These are the 15 clinical measurements in the adjustment set. Age, admission year, and time from ICU admission to T0 were complete. The primary models used median imputation with missingness indicators. The multiple-imputation sensitivity analysis is detailed in Supplementary Methods S5 and Table S9.

**Supplementary Table S2A. Actual eligible aspirin in the 24 hours before T0.**

| **Pre-index record** | **No early aspirin, n (%)** | **Early aspirin, n (%)** |
| --- | --- | --- |
| Absent | 27,773 (98.3%) | 2,316 (63.5%) |
| Present | 469 (1.7%) | 1,330 (36.5%) |

Early aspirin means an eligible administration within 24 hours after T0. Absence of a record does not establish aspirin-naïve status.

**Supplementary Table S2B. Mortality stratified by recorded pre-index aspirin.**

| **Outcome** | **Recorded history** | **Interval after T0** | **Patients** | **Deaths** | **HR (95% CI)** | **P value** |
| --- | --- | --- | --- | --- | --- | --- |
| In-hospital | No | 0 to <24 h | 30,089 | 4,925 | 0.46 (0.26–0.79) | 0.005 |
| In-hospital | No | 24 to <72 h | 30,089 | 4,925 | 0.64 (0.50–0.83) | <0.001 |
| In-hospital | No | 72 to <168 h | 30,089 | 4,925 | 0.79 (0.65–0.96) | 0.019 |
| In-hospital | No | 168 h to 28 days | 30,089 | 4,925 | 1.01 (0.88–1.16) | 0.889 |
| ICU | No | 0 to <24 h | 30,089 | 3,566 | 0.46 (0.27–0.80) | 0.006 |
| ICU | No | 24 to <72 h | 30,089 | 3,566 | 0.67 (0.52–0.87) | 0.002 |
| ICU | No | 72 to <168 h | 30,089 | 3,566 | 0.85 (0.68–1.06) | 0.160 |
| ICU | No | 168 h to 28 days | 30,089 | 3,566 | 1.16 (0.96–1.40) | 0.129 |
| In-hospital | Yes | 0 to <24 h | 1,799 | 283 | 0.40 (0.17–0.97) | 0.043 |
| In-hospital | Yes | 24 to <72 h | 1,799 | 283 | 0.43 (0.24–0.76) | 0.004 |
| In-hospital | Yes | 72 to <168 h | 1,799 | 283 | 0.85 (0.48–1.49) | 0.566 |
| In-hospital | Yes | 168 h to 28 days | 1,799 | 283 | 1.04 (0.52–2.05) | 0.915 |
| ICU | Yes | 0 to <24 h | 1,799 | 226 | 0.40 (0.16–0.97) | 0.043 |
| ICU | Yes | 24 to <72 h | 1,799 | 226 | 0.44 (0.24–0.83) | 0.011 |
| ICU | Yes | 72 to <168 h | 1,799 | 226 | 0.72 (0.37–1.41) | 0.341 |
| ICU | Yes | 168 h to 28 days | 1,799 | 226 | 1.39 (0.49–3.92) | 0.537 |

Global interaction P=0.122 for in-hospital and P=0.393 for ICU mortality. Adjustment follows Supplementary Methods S1; MAP is linear in these models.

**Supplementary Table S2C. Mortality with no recorded eligible aspirin since hospital admission.**

| **Outcome** | **Interval after T0** | **Patients** | **Deaths** | **HR (95% CI)** | **P value** |
| --- | --- | --- | --- | --- | --- |
| In-hospital | 0 to <24 h | 29,677 | 4,821 | 0.38 (0.20–0.70) | 0.002 |
| In-hospital | 24 to <72 h | 29,677 | 4,821 | 0.60 (0.46–0.79) | <0.001 |
| In-hospital | 72 to <168 h | 29,677 | 4,821 | 0.79 (0.64–0.96) | 0.019 |
| In-hospital | 168 h to 28 days | 29,677 | 4,821 | 0.98 (0.85–1.13) | 0.785 |
| ICU | 0 to <24 h | 29,677 | 3,501 | 0.38 (0.20–0.71) | 0.002 |
| ICU | 24 to <72 h | 29,677 | 3,501 | 0.63 (0.48–0.82) | <0.001 |
| ICU | 72 to <168 h | 29,677 | 3,501 | 0.86 (0.68–1.08) | 0.182 |
| ICU | 168 h to 28 days | 29,677 | 3,501 | 1.15 (0.94–1.40) | 0.171 |

Exclusion of 2,211 patients with admission-to-T0 administration left 29,677 patients. This broader inpatient lookback is not verified outpatient medication history.

**Supplementary Table S3. Hemoglobin decline and PRBC administration.**

| **Outcome** | **Patients** | **No early aspirin events/N** | **Early aspirin events/N** | **Adjusted RR (95% CI)** | **P value** |
| --- | --- | --- | --- | --- | --- |
| Hemoglobin decline ≥2 g/dL, 0–72 h | 16,410 | 4,350/14,033 | 963/2,377 | 1.10 (1.03–1.17) | 0.004 |
| Incident hemoglobin decline ≥2 g/dL, 24–72 h | 9,947 | 1,756/8,630 | 257/1,317 | 1.05 (0.91–1.21) | 0.502 |
| Any PRBC, 0–72 h | 31,888 | 7,042/28,242 | 717/3,646 | 0.88 (0.82–0.96) | 0.003 |
| Any PRBC, 24–72 h | 22,693 | 1,727/19,828 | 221/2,865 | 0.84 (0.70–1.00) | 0.046 |

Concurrent windows overlap treatment ascertainment. A qualifying hemoglobin decline preceded the first dose in 606 early recipients. Incident 24–72-hour analyses require the relevant event to be absent during 0–24 hours and continued observation beyond 24 hours; the hemoglobin sample additionally requires observed baseline and both follow-up measurement windows. These are different selected populations.

**Supplementary Table S4. Selection into the landmark sample.**

| **Stage or event** | **Patients** | **% of primary cohort** |
| --- | --- | --- |
| Primary analytic cohort | 31,888 | 100.0% |
| Eligible beyond both hospital and ICU 24-hour landmarks | 28,036 | 87.9% |
| Excluded from landmark analysis | 3,852 | 12.1% |
| Hospital death within 24 hours | 698 | 2.2% |
| Hospital discharge alive within 24 hours | 142 | 0.4% |
| ICU death within 24 hours | 689 | 2.2% |
| ICU discharge without ICU death within 24 hours | 3,149 | 9.9% |

Early death and discharge categories overlap and must not be summed. After matching, the analysis included 4,922 patients (2,461 pairs).

**Supplementary Table S5A. T0 relative to ICU admission.**

| **Time after ICU admission** | **Patients** | **%** |
| --- | --- | --- |
| At ICU admission | 16,845 | 52.8% |
| >0–6 hours | 10,754 | 33.7% |
| >6–24 hours | 1,613 | 5.1% |
| >24–48 hours | 783 | 2.5% |
| >48 hours | 1,893 | 5.9% |

**Supplementary Table S5B. Mortality restricted to T0 at ICU admission.**

| **Outcome** | **Interval after T0** | **Patients** | **Deaths** | **HR (95% CI)** | **P value** |
| --- | --- | --- | --- | --- | --- |
| In-hospital | 0 to <24 h | 16,845 | 2,830 | 0.43 (0.22–0.84) | 0.014 |
| In-hospital | 24 to <72 h | 16,845 | 2,830 | 0.54 (0.40–0.74) | <0.001 |
| In-hospital | 72 to <168 h | 16,845 | 2,830 | 0.81 (0.65–1.03) | 0.083 |
| In-hospital | 168 h to 28 days | 16,845 | 2,830 | 1.04 (0.87–1.25) | 0.660 |
| ICU | 0 to <24 h | 16,845 | 1,968 | 0.44 (0.22–0.85) | 0.014 |
| ICU | 24 to <72 h | 16,845 | 1,968 | 0.55 (0.40–0.77) | <0.001 |
| ICU | 72 to <168 h | 16,845 | 1,968 | 0.88 (0.67–1.17) | 0.382 |
| ICU | 168 h to 28 days | 16,845 | 1,968 | 1.23 (0.96–1.59) | 0.105 |

**Supplementary Table S5C. Mortality with qualifying Sepsis-3 SOFA by T0.**

| **Outcome** | **Interval after T0** | **Patients** | **Deaths** | **HR (95% CI)** | **P value** |
| --- | --- | --- | --- | --- | --- |
| In-hospital | 0 to <24 h | 6,972 | 1,573 | 0.54 (0.28–1.03) | 0.061 |
| In-hospital | 24 to <72 h | 6,972 | 1,573 | 0.62 (0.44–0.88) | 0.008 |
| In-hospital | 72 to <168 h | 6,972 | 1,573 | 0.82 (0.63–1.06) | 0.135 |
| In-hospital | 168 h to 28 days | 6,972 | 1,573 | 0.76 (0.60–0.96) | 0.022 |
| ICU | 0 to <24 h | 6,972 | 1,213 | 0.52 (0.27–0.99) | 0.047 |
| ICU | 24 to <72 h | 6,972 | 1,213 | 0.62 (0.43–0.90) | 0.012 |
| ICU | 72 to <168 h | 6,972 | 1,213 | 0.80 (0.59–1.08) | 0.141 |
| ICU | 168 h to 28 days | 6,972 | 1,213 | 0.82 (0.61–1.10) | 0.179 |

Qualifying SOFA was recorded after T0 in 24,916/31,888 patients (78.1%); 6,972 remained in this restriction. The primary retrospective T0 definition was unchanged. A qualifying SOFA by T0 is distinct from the strict pre-index SOFA covariate.

**Supplementary Table S6A. Observed baseline platelet subgroups.**

| **Platelet count** | **No early aspirin** | **Early aspirin** | **Total** |
| --- | --- | --- | --- |
| <100×10^9/L | 2,044 | 216 | 2,260 |
| ≥100×10^9/L | 12,452 | 2,180 | 14,632 |

An observed baseline platelet count was available for 16,892 patients. Missing: 14,996 (47.0%).

**Supplementary Table S6B. Exploratory mortality associations by platelet count.**

| **Outcome** | **Platelet count** | **Interval after T0** | **Patients** | **Deaths** | **HR (95% CI)** | **P value** |
| --- | --- | --- | --- | --- | --- | --- |
| In-hospital | ≥100×10^9/L | 0 to <24 h | 14,632 | 2,221 | 0.70 (0.41–1.19) | 0.189 |
| In-hospital | ≥100×10^9/L | 24 to <72 h | 14,632 | 2,221 | 0.77 (0.58–1.01) | 0.061 |
| In-hospital | ≥100×10^9/L | 72 to <168 h | 14,632 | 2,221 | 0.97 (0.78–1.20) | 0.752 |
| In-hospital | ≥100×10^9/L | 168 h to 28 days | 14,632 | 2,221 | 0.94 (0.78–1.14) | 0.549 |
| ICU | ≥100×10^9/L | 0 to <24 h | 14,632 | 1,643 | 0.70 (0.41–1.20) | 0.194 |
| ICU | ≥100×10^9/L | 24 to <72 h | 14,632 | 1,643 | 0.74 (0.55–0.99) | 0.042 |
| ICU | ≥100×10^9/L | 72 to <168 h | 14,632 | 1,643 | 0.90 (0.70–1.16) | 0.426 |
| ICU | ≥100×10^9/L | 168 h to 28 days | 14,632 | 1,643 | 1.06 (0.84–1.34) | 0.640 |
| In-hospital | <100×10^9/L | 0 to <24 h | 2,260 | 660 | 0.23 (0.03–1.71) | 0.152 |
| In-hospital | <100×10^9/L | 24 to <72 h | 2,260 | 660 | 0.51 (0.25–1.03) | 0.061 |
| In-hospital | <100×10^9/L | 72 to <168 h | 2,260 | 660 | 0.38 (0.20–0.73) | 0.004 |
| In-hospital | <100×10^9/L | 168 h to 28 days | 2,260 | 660 | 0.45 (0.29–0.72) | <0.001 |
| ICU | <100×10^9/L | 0 to <24 h | 2,260 | 497 | 0.24 (0.03–1.80) | 0.166 |
| ICU | <100×10^9/L | 24 to <72 h | 2,260 | 497 | 0.59 (0.30–1.19) | 0.139 |
| ICU | <100×10^9/L | 72 to <168 h | 2,260 | 497 | 0.43 (0.21–0.88) | 0.020 |
| ICU | <100×10^9/L | 168 h to 28 days | 2,260 | 497 | 0.55 (0.29–1.03) | 0.062 |

Global interaction P<0.001 for in-hospital and P=0.020 for ICU mortality. This selected exploratory subgroup does not establish sepsis-induced thrombocytopenia or DIC. Analyses were not adjusted for multiplicity.

**Supplementary Table S7. New invasive ventilation.**

| **Outcome** | **Patients** | **No early aspirin events/N** | **Early aspirin events/N** | **Adjusted RR (95% CI)** | **P value** |
| --- | --- | --- | --- | --- | --- |
| New invasive ventilation, 0–72 h | 24,363 | 9,122/21,494 | 1,192/2,869 | 0.99 (0.94–1.04) | 0.723 |
| New invasive ventilation, 24–72 h | 12,276 | 847/10,731 | 107/1,545 | 0.82 (0.65–1.05) | 0.116 |

Both samples exclude baseline ventilatory support of any type. The 24–72-hour sample also excludes earlier invasive ventilation and requires continued observation at 24 hours. The outcome is not a validated ARDS phenotype.

**Supplementary Table S8A. Continuous-time MSM weight diagnostics.**

| **Diagnostic** | **Value** |
| --- | --- |
| Risk-day records | 150,256 |
| Initiations through 28 days | 4,761 |
| Initiations at or after 72 hours | 378 |
| Raw weight mean | 0.983 |
| Raw weight SD | 0.492 |
| Raw minimum | 0.059 |
| Raw 1st percentile | 0.320 |
| Raw median | 0.899 |
| Raw 99th percentile | 2.264 |
| Raw maximum | 32.499 |
| Risk-record ESS raw | 120,120 |
| Risk-record ESS truncated | 142,609 |
| Records truncated | 3,006 |
| Denominator 24-hour initiation probability below 0.01 | 90,288 |

MSM cohort: 31,885, excluding three doses at T0. Both Poisson rate models converged with no aliased coefficients. Percentiles and effective sample sizes refer to untreated risk-day endpoint records, not unique patients. No probability clipping was used. Supplementary Figure S2 displays these untreated risk-day endpoint weights before and after truncation.

**Supplementary Table S8B. MSM estimates by weight truncation.**

| **Outcome** | **Weights** | **Interval after T0** | **Patients** | **Deaths** | **HR (95% CI)** | **P value** |
| --- | --- | --- | --- | --- | --- | --- |
| In-hospital | Raw | 0 to <24 h | 31,885 | 5,208 | 0.70 (0.17–2.83) | 0.619 |
| In-hospital | Raw | 24 to <72 h | 31,885 | 5,208 | 0.57 (0.38–0.87) | 0.009 |
| In-hospital | Raw | 72 to <168 h | 31,885 | 5,208 | 0.79 (0.61–1.02) | 0.067 |
| In-hospital | Raw | 168 h to 28 days | 31,885 | 5,208 | 1.00 (0.83–1.21) | 0.996 |
| In-hospital | 1st/99th | 0 to <24 h | 31,885 | 5,208 | 0.38 (0.22–0.68) | <0.001 |
| In-hospital | 1st/99th | 24 to <72 h | 31,885 | 5,208 | 0.55 (0.42–0.72) | <0.001 |
| In-hospital | 1st/99th | 72 to <168 h | 31,885 | 5,208 | 0.90 (0.74–1.09) | 0.276 |
| In-hospital | 1st/99th | 168 h to 28 days | 31,885 | 5,208 | 1.02 (0.89–1.18) | 0.747 |
| In-hospital | 2.5th/97.5th | 0 to <24 h | 31,885 | 5,208 | 0.47 (0.30–0.75) | 0.001 |
| In-hospital | 2.5th/97.5th | 24 to <72 h | 31,885 | 5,208 | 0.61 (0.49–0.75) | <0.001 |
| In-hospital | 2.5th/97.5th | 72 to <168 h | 31,885 | 5,208 | 0.89 (0.76–1.04) | 0.143 |
| In-hospital | 2.5th/97.5th | 168 h to 28 days | 31,885 | 5,208 | 1.00 (0.88–1.12) | 0.955 |
| ICU | Raw | 0 to <24 h | 31,885 | 3,792 | 0.72 (0.18–2.89) | 0.640 |
| ICU | Raw | 24 to <72 h | 31,885 | 3,792 | 0.63 (0.41–0.97) | 0.036 |
| ICU | Raw | 72 to <168 h | 31,885 | 3,792 | 0.84 (0.63–1.12) | 0.242 |
| ICU | Raw | 168 h to 28 days | 31,885 | 3,792 | 0.99 (0.77–1.27) | 0.944 |
| ICU | 1st/99th | 0 to <24 h | 31,885 | 3,792 | 0.39 (0.22–0.69) | 0.001 |
| ICU | 1st/99th | 24 to <72 h | 31,885 | 3,792 | 0.59 (0.45–0.78) | <0.001 |
| ICU | 1st/99th | 72 to <168 h | 31,885 | 3,792 | 0.95 (0.76–1.19) | 0.679 |
| ICU | 1st/99th | 168 h to 28 days | 31,885 | 3,792 | 1.10 (0.91–1.33) | 0.318 |
| ICU | 2.5th/97.5th | 0 to <24 h | 31,885 | 3,792 | 0.48 (0.31–0.76) | 0.002 |
| ICU | 2.5th/97.5th | 24 to <72 h | 31,885 | 3,792 | 0.63 (0.51–0.79) | <0.001 |
| ICU | 2.5th/97.5th | 72 to <168 h | 31,885 | 3,792 | 0.94 (0.78–1.13) | 0.511 |
| ICU | 2.5th/97.5th | 168 h to 28 days | 31,885 | 3,792 | 1.09 (0.93–1.27) | 0.314 |

The initiation process includes all observed first doses during up to 28 days of untreated ICU observation, including those at or after 72 hours. First-interval estimates are sensitive to truncation.

**Supplementary Table S8C. Standardized initiation-process balance.**

| **Covariate** | **Day 0 raw** | **Day 0 trunc.** | **Day 1 raw** | **Day 1 trunc.** | **Day 2 raw** | **Day 2 trunc.** | **Day ≥3 raw** | **Day ≥3 trunc.** |
| --- | --- | --- | --- | --- | --- | --- | --- | --- |
| Age | 0.165 | 0.199 | 0.077 | 0.244 | 0.257 | 0.242 | 0.020 | 0.135 |
| SOFA score | -0.068 | 0.045 | -0.111 | -0.115 | -0.089 | -0.031 | 0.032 | 0.116 |
| Platelet count | -0.044 | -0.047 | -0.040 | -0.005 | 0.038 | 0.054 | -0.120 | -0.115 |
| INR | -0.020 | -0.027 | -0.110 | -0.106 | -0.140 | -0.094 | 0.088 | 0.129 |
| Lactate | -0.068 | -0.045 | -0.011 | 0.036 | -0.029 | 0.016 | 0.081 | 0.046 |
| Prior-day hemoglobin | -0.021 | 0.009 | 0.042 | 0.048 | -0.305 | -0.235 | -0.018 | -0.020 |
| Prior-day platelet count | -0.044 | -0.047 | -0.108 | -0.060 | 0.216 | 0.149 | 0.047 | 0.038 |
| Prior-day inr | -0.020 | -0.027 | 0.005 | -0.027 | 0.156 | 0.054 | 0.034 | 0.005 |
| Prior-day lactate | -0.068 | -0.045 | 0.125 | 0.024 | -0.097 | -0.081 | 0.048 | 0.004 |
| Prior-day creatinine | 0.052 | 0.074 | -0.029 | 0.060 | 0.081 | 0.152 | 0.045 | 0.093 |
| Prior-day mean arterial pressure (map) | 0.019 | -0.006 | 0.143 | 0.152 | 0.055 | 0.058 | 0.082 | 0.083 |
| Prior-day sofa score | -0.068 | 0.045 | 0.110 | 0.097 | 0.114 | 0.158 | -0.080 | -0.057 |
| Prior-day ventilatory support | -0.126 | -0.094 | 0.001 | 0.004 | -0.397 | -0.315 | -0.110 | -0.151 |
| Prior-day vasopressor use | -0.033 | -0.004 | 0.061 | 0.068 | 0.058 | 0.085 | -0.115 | -0.089 |
| Prior-day PRBC | -0.132 | -0.082 | -0.040 | -0.087 | -0.224 | -0.230 | 0.013 | 0.027 |
| Recorded pre-index aspirin | 0.116 | 0.335 | 0.060 | 0.226 | -0.117 | 0.031 | -0.044 | 0.105 |

Signed standardized differences compare weighted observed initiations with marginal-intensity-weighted untreated risk time. Trunc. denotes 1st/99th-percentile truncation. This is not a binary daily treatment SMD. Residual differences, including a maximum absolute truncated difference of 0.335, limit causal interpretation.

Supplementary Table S9. Time-dependent mortality estimates from the original and multiple-imputation models.

The original model used median imputation with missingness indicators. For the MICE analysis, 15 pre-index clinical measurements were imputed in 20 data sets, and missingness indicators were omitted. The cohort, exact-time aspirin exposure, outcome definitions, and other baseline covariates were unchanged. Intervals are measured from T0; values are HR (95% CI).

| Outcome | Interval after T0 | Original HR (95% CI) | MICE HR (95% CI) |
| --- | --- | --- | --- |
| In-hospital | 0 to <24 h | 0.48 (0.31–0.75) | 0.50 (0.32–0.79) |
| In-hospital | 24 to <72 h | 0.63 (0.51–0.78) | 0.66 (0.53–0.82) |
| In-hospital | 72 to <168 h | 0.87 (0.74–1.02) | 0.90 (0.77–1.06) |
| In-hospital | 168 h to 28 days | 0.94 (0.82–1.07) | 0.98 (0.85–1.12) |
| ICU | 0 to <24 h | 0.49 (0.31–0.77) | 0.51 (0.32–0.80) |
| ICU | 24 to <72 h | 0.65 (0.52–0.80) | 0.67 (0.54–0.84) |
| ICU | 72 to <168 h | 0.91 (0.76–1.10) | 0.93 (0.77–1.13) |
| ICU | 168 h to 28 days | 1.07 (0.90–1.28) | 1.10 (0.91–1.32) |

# Supplementary Figure S1

Figure S1. Absolute standardized mean differences for baseline missingness indicators before and after landmark matching. The dashed line marks 0.10.

# Supplementary Figure S2

Figure S2. Distribution of stabilized continuous-time initiation weights before and after 1st/99th-percentile truncation. The horizontal axis and record counts are shown on logarithmic scales to display both tails. Dashed vertical lines mark the truncation limits (0.320 and 2.264). The analysis included 150,256 untreated risk-day endpoint records, not 150,256 unique patients. The largest raw weight was 32.499; truncation affected 3,006 records (2.0%).
